# A comparison of large language model-generated and published perioperative neurocognitive disorder recommendations: a cross-sectional web-based analysis

**DOI:** 10.1016/j.bja.2025.01.001

**Published:** 2025-02-07

**Authors:** Sarah Saxena, Odmara L. Barreto Chang, Melanie Suppan, Basak Ceyda Meco, Susana Vacas, Finn Radtke, Idit Matot, Arnout Devos, Mervyn Maze, Mia Gisselbaek, Joana Berger-Estilita

**Affiliations:** 1 Department of Surgery, Research Institute for Health Sciences and Technology, University of Mons, Mons, Belgium; 2 Department of Anesthesiology, Helora, Mons, Belgium; 3 Department of Anesthesia and Perioperative Care, University of California San Francisco, San Francisco, CA, USA; 4 Division of Anesthesiology, Department of Anesthesiology, Clinical Pharmacology, Intensive Care and Emergency Medicine, Geneva University Hospitals and Faculty of Medicine, Geneva, Switzerland; 5 Department of Anaesthesia and Intensive Care, Ankara University Faculty of Medicine, Ankara, Turkey; 6 Ankara University Brain Research Center (BAUM), Ankara, Turkey; 7 Department of Anesthesiology, Critical Care, and Pain Medicine, Massachusetts General Hospital, Harvard Medical School, Boston, MA, USA; 8 Department of Anaesthesia and Intensive Care, Hospital of Nykøbing Falster, University of Southern Denmark, Odense, Denmark; 9 Division of Anesthesia, Intensive Care, and Pain Management, Tel-Aviv Medical Center, Tel-Aviv University, Tel-Aviv, Israel; 10 ETH AI Center, Swiss Federal Institute of Technology Zurich (ETH Zurich), Zurich, Switzerland; 11 Unit of Development and Research in Medical Education (UDREM), Faculty of Medicine, University of Geneva, Geneva, Switzerland; 12 Institute for Medical Education, University of Bern, Bern, Switzerland; 13 CINTESIS@RISE, Centre for Health Technology and Services Research, Faculty of Medicine, University of Porto, Porto, Portugal

**Keywords:** artificial intelligence (AI), ChatGPT-4, clinical guidelines, Gemini, large language models (LLM), patient outcomes, perioperative neurocognitive disorders (PND)

## Abstract

**Background:**

Perioperative neurocognitive disorders (PNDs) are common complications after surgery and anaesthesia, particularly in older adults, leading to increased morbidity, mortality, and healthcare costs. Therefore, major medical societies have developed recommendations for the prevention and treatment of PNDs. Our study evaluated the reliability of large language models, specifically ChatGPT-4 and Gemini, in generating recommendations for PND management and comparing them with published guidelines.

**Methods:**

We conducted an online cross-sectional web-based analysis over 48 h in June 2024. Artificial intelligence (AI)-generated recommendations were produced in six different locations across five countries (Switzerland, Belgium, Turkey, Canada, and the East and West Coasts of the USA). The English prompt *‘a table of a bundle of care for perioperative neurocognitive disorders’* was entered into ChatGPT-4 and Gemini, generating tables evaluated by independent reviewers. The primary outcomes were the Total Disagreement Score (TDS) and Quality Assessment of Medical Artificial Intelligence (QAMAI), which compared AI-generated recommendations with published guidelines.

**Results:**

The study generated 14 tables, with TDS and QAMAI scores showing similar results for ChatGPT-4 and Gemini (2 [1–3] *vs* 2 [2–3], *P*=0.636 and 4 [4–4] *vs* 4 [3–4], *P*=0.424, respectively). AI-generated recommendations aligned well with published guidelines, with the highest alignment observed in ChatGPT-4-generated recommendations. No complete agreement with guidelines was achieved, and lack of cited sources was a noted limitation.

**Conclusions:**

Large language models can generate perioperative neurocognitive disorder recommendations that align closely with published guidelines. However, further validation and integration of clinician feedback are required before clinical application.

**Editor's key points**

• Writing recommendations for perioperative management is a resource-intensive process, requiring systematic review of the literature and consensus meetings among experts.
• Generative artificial intelligence (AI) systems can facilitate this process through rapid scanning and sorting of published information.
• In this study, two generative AI systems (ChatGPT-4 and Gemini) were prompted to create recommendations for preventing and treating postoperative delirium. These recommendations were compared with two society-generated published guidelines.
• AI-generated recommendations aligned well with published guidelines, although complete agreement was not achieved, and lack of cited sources was a noted limitation.
• Although not proven to be a replacement for the traditional process, AI-generated recommendations might streamline creation and revision of perioperative management guidelines.

Perioperative neurocognitive disorders (PNDs) are common complications after anaesthesia and surgery,^1^, ^2^, ^3^, ^4^ leading to increased morbidity, mortality, length of hospital stay, and healthcare costs.^3^^,^^5^, ^6^, ^7^, ^8^ Risk factors include older age, lower educational attainment, and baseline cognitive impairment.^1^^,^^3^^,^^9^ Because of the significant detrimental outcomes, major medical societies around the world have developed guidelines based on expert consensus about the evidence for the prevention and treatment of PND.^2^^,^^10^, ^11^, ^12^, ^13^, ^14^ Their recommendations often include perioperative care bundles (small sets of evidence-based practices) to minimise postoperative delirium and long-term cognitive decline,^10^^,^^14^ using a multidisciplinary longitudinal approach encompassing nonpharmacological interventions and avoiding deliriogenic medications.^2^^,^^10^, ^11^, ^12^, ^13^, ^14^

Recently, generative artificial intelligence (GenAI) has expanded its presence in medical applications. AI-based systems can perform problem-solving tasks to facilitate clinical decisions based on the data it is fed.^15^ Traditionally, most AI-based systems have been highly domain-specific, where systems that provide a discriminative decision are the most common. Recent breakthroughs in GenAI with very deep artificial neural networks (deep learning) and a natural language interface have made these AI systems more domain-agnostic, interactive, and aligned with human intent.^16^ In addition, AI is updating at a faster rate than evidenced-based, expert-generated guidelines.^17^

Despite the potential advantages of using modern generative AI in clinical practice, its limitations have yet to be fully explored. Large language models (LLMs) are pioneering technologies transforming how we interact with information across various domains. They are being introduced into medical education,^18^ and recent medical graduates increasingly rely on these tools.^19^^,^^20^

Some are advocating for the integration of AI to improve and streamline anaesthetic practice.^21^^,^^22^ This could involve focusing on areas such as depth of anaesthesia titration and monitoring, prediction of events and risks, ultrasound guidance, pain management, and optimisation of perioperative logistics.^23^, ^24^, ^25^ Integrating LLMs such as ChatGPT-4 and Gemini into medical practice may offer a new avenue for rapid access to information that can ultimately improve patient outcomes. However, a gap exists in the literature regarding the accuracy of LLMs such as ChatGPT-4 and Gemini in developing recommendations for care.

We aimed to compare AI-generated recommendations with currently published guidelines for the prevention of PNDs and assess the potential use of AI systems in developing recommendations.^10^, ^11^, ^12^, ^13^, ^14^

## Methods

### Ethics

The Cantonal Ethics Committee of Bern (BASEC-number: Req-2024-00531) waived ethics approval for the study on April 12, 2024, because it did not involve human participants. The study adhered to the Declaration of Helsinki,^26^ and researchers followed the Data Protection Acts of their respective academic institutions. The study followed the Strengthening the Reporting of Observational Studies in Epidemiology (STROBE) reporting guideline.^27^

### Study design and setting

This study is a cross-sectional web-based analysis conducted over 48 h in June 2024. The AI recommendations were generated in six locations in five countries (USA [East and West Coasts], Canada, Switzerland, Belgium, and Turkey). Different countries were included to represent real-world coverage. Differences in AI-generated recommendations may arise as a result of the interaction between the model's inherent cultural biases and the way users from different linguistic and cultural backgrounds formulate their queries.^28^ This can lead to variations in output that appear location-specific, although the underlying model remains consistent globally.^29^^,^^30^

### AI model data generation

During 48 h (June 5–7, 2024), the following English prompt, ‘*a table of a bundle of care for perioperative neurocognitive disorders*’, was used to generate tables in ChatGPT-4 (available at https://openai.com/blog/chatgpt from OpenAI, San Francisco, CA, USA) and Gemini (available at https://gemini.google.com/from Alphabet Inc., Mountain View, CA, USA). The tables were generated in six different locations (Belgium, Switzerland, USA [East and West Coasts], Turkey, and Canada). Each request was entered individually in a new dialogue box. In total, we aimed to generate 12 tables by entering prompt into ChatGPT-4 and Gemini in each of the six locations.

### Sample size

The sample size (six locations, two published guidelines, and two GenAI tools) was selected to balance geographical representation and resource constraints. The countries were chosen to represent a diverse range of healthcare environments across North America and Europe. This selection aimed to capture different healthcare policies, practices, and standards, thus providing a broad perspective on perioperative brain health approaches. The Brain Health Initiative (BHI)^10^^,^^13^ and the Safe Brain Initiative (SBI)^11^ guidelines were selected based on a literature review that considered their availability, broad geographical scope, and widespread application across North America and Europe. Finally, we chose OpenAI ChatGPT-4 and Google Gemini, as they are the two most widely used GenAI chatbots, with market shares of 60% and 14%, respectively, in the USA (https://firstpagesage.com/reports/top-generative-ai-chatbots/). Their selection ensured that our study captured insights from the most prevalent AI tools currently used for clinical and academic purposes. This combination of locations, guidelines, and AI tools was intentionally designed to provide a balanced representation of varied healthcare contexts, established standards in perioperative care, and leading AI technologies while keeping the project manageable and focused.

### Classification

The responses generated by each LLM were collected in a Google Document file (Alphabet Inc.) and blinded for origin and AI model by MG, who was not involved in data analysis. A panel of two researchers evaluated the blinded PND recommendations, with a third researcher resolving disagreements. The evaluators were experts in PND, participated in the SBI and BHI recommendations^10^, ^11^, ^12^, ^13^, ^14^ (Supplementary material 1), and authored relevant work on perioperative brain health.^1^, ^2^, ^3^, ^4^ Evaluators rated the proposed recommendations on the basis of the following: a multidisciplinary team for the prevention, diagnosis, and management of PND; preoperative risk disclosure; preoperative identification of risk factors; baseline neurocognitive screening; nonpharmacological measures for PND prevention; benzodiazepines use/no use; monitoring and titration of the depth of anaesthesia; assessment of perioperative nociception (monitored opioid use); and postoperative neurocognitive screening.^10^, ^11^, ^12^, ^13^, ^14^ Two different, previously validated scoring systems were used to rate the tables.^31^^,^^32^

### Total Disagreement Score

The first panel (BCM and JBE, with SV for disagreements) rated the LLM answers using the Total Disagreement Score (TDS)^28^ system for medical content. The TDS system rated LLM answers on a scale from 0 (complete agreement) to 12 (complete disagreement). For the study, this score was derived from partial scores ranging from 0 (no disagreement) to 3 (major disagreement) across four domains: *preoperative management*; *intraoperative management*; *postoperative management*; and *other concerns*. The highest value of disagreement (complete disagreement being 3) was retained for each domain and added up for the total TDS score.

### Quality Assessment of Medical Artificial Intelligence

The second panel (OLBC, SS, with FR for disagreements) rated the LLM answers using the Quality Assessment of Medical Artificial Intelligence (QAMAI) instrument.^32^ This instrument indicates the level of agreement with the LLM answer from 1 (strongly disagree) to 5 (strongly agree) across six domains: a*ccuracy*, c*larity*, *relevance*, c*ompleteness*, *provision of sources*, and *usefulness.*

### Agreement definitions

For the purposes of this study, before analysis, the levels of agreement between the evaluators' ratings of AI-generated recommendations and the published guidelines were defined as: **overall complete agreement**, when the TDS score is 0, and the QAMAI score is 5 across all domains; **overall agreement**, when the TDS score is between 1 and 3, and the QAMAI score is 4 or higher across all domains; and **overall disagreement**, when the TDS score is 4 or higher, and the QAMAI score is 3 or lower in any domain.

### Data collection and analysis

A single TDS score for each question and a single QAMAI evaluation for each pair domain/answer were generated for each of the AI-generated tables. The TDS and QAMAI scores were collected in a Google Sheets file (Alphabet Inc.). Subsequently, LG unblinded them for statistical analysis.

### Risk of bias assessment

JBE and SS created a ‘Risk of bias’ table to systematically compare recommendations from the BHI and SBI guidelines with those proposed by LLM AI systems (Table 1). This table followed the Cochrane Collaboration's recommended tool for assessing the risk of bias,^33^ which uses a domain-based evaluation where critical assessments are made separately for different domains.

**Table 1:**
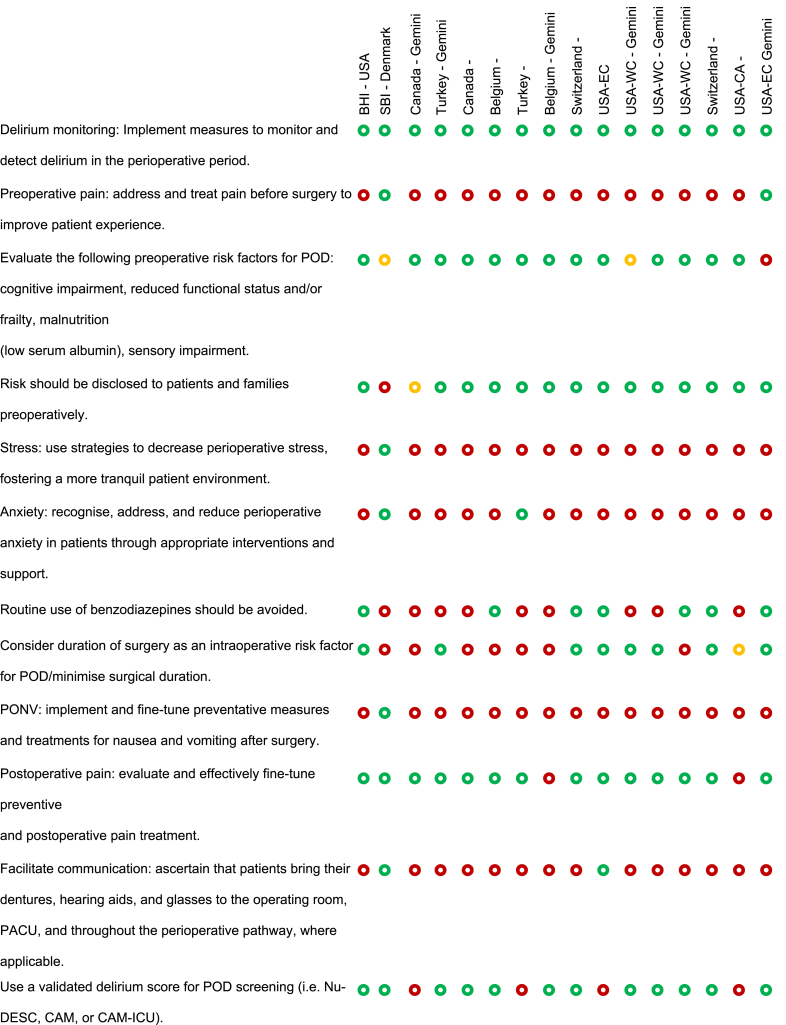

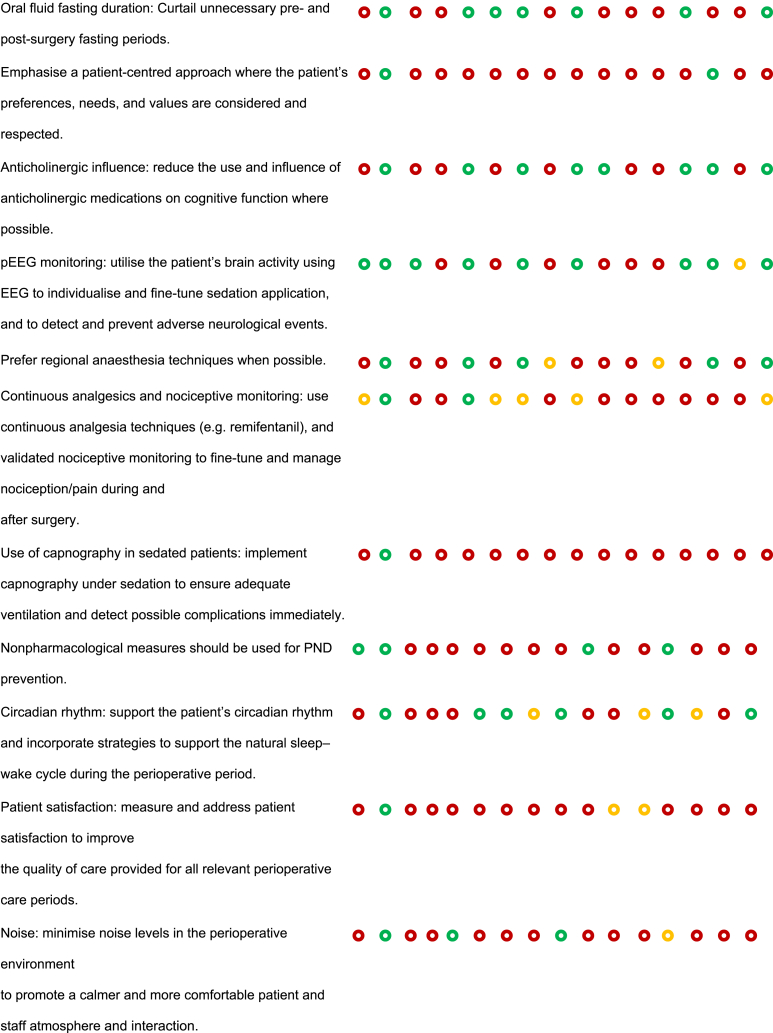

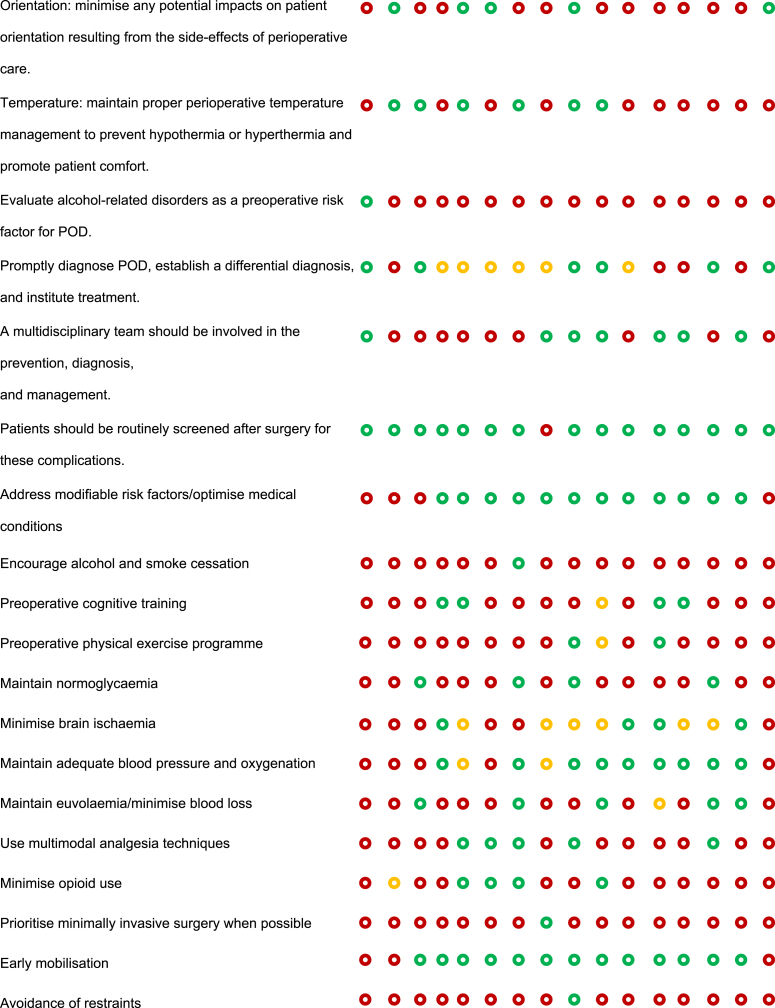

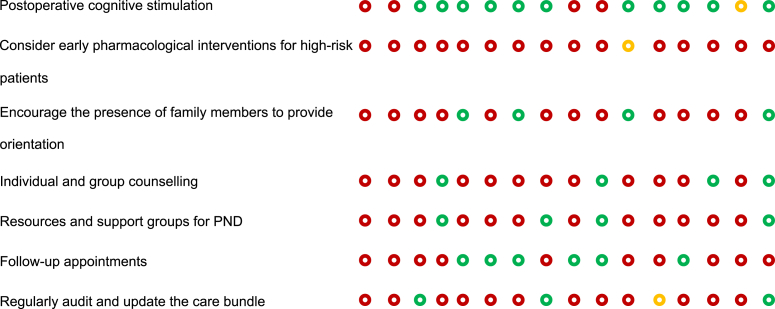
Risk of bias assessment comparing guideline recommendations (BHI/USA and SBI/EU) with AI recommendations. The table compares the inclusion of specific perioperative neurocognitive disorder (PND) care recommendations across different sources: Brain Health Initiative (BHI) in the USA, Safe Brain Initiative (SBI) in Denmark, and AI-generated care bundles using ChatGPT-4 and Gemini models across various countries and regions. Each recommendation is marked as either mentioned (), partially mentioned (), or not mentioned (). Recommendations that are not included in the BHI or SBI guidelines but generated by the AI models are highlighted in **bold**. POD, postoperative delirium; PONV, postoperative nausea and vomiting.

The aim was to assess how well the AI recommendations aligned with published guidelines and to highlight areas where AI may need further improvement or where it may offer novel insights. This approach provided a comprehensive evaluation of potential biases that could influence both the recommendations and their implementation.

To begin, SS and JBE compiled a list of relevant perioperative care recommendations from the BHI and SBI guidelines,^10^, ^11^, ^12^, ^13^, ^14^ which served as the benchmark. A comparison framework was established where each recommendation from the BHI and SBI guidelines was listed alongside the corresponding AI-generated recommendations. This framework allowed for a direct comparison to determine whether the AI-generated recommendations align with, partially align with, or diverge from the published guidelines. For each recommendation, symbols were used to indicate whether it was mentioned (green circle), partially mentioned (yellow circle), or not mentioned (red circle) by the BHI, SBI, and AI. Any additional recommendations outside the BHI and SBI guidelines proposed by AI were noted in bold, highlighting unique contributions or suggestions made by AI. The information was compiled into a table format, ensuring that each recommendation was aligned with the respective guideline or AI output. The use of visual symbols and bold text helped quickly to identify areas of agreement, partial agreement, and divergence.

A scoring system was implemented to facilitate quantitative comparisons between the recommendations. In this system, items that were mentioned received 1 point, items that were partially mentioned received 0.5 points, and items that were not mentioned received 0 points. This scoring allowed for a more objective evaluation of how closely AI-generated recommendations matched those of the published guidelines.

OLBC and BCM reviewed and validated the table to ensure the accuracy of the comparisons and the appropriateness of the recommendations. This validation step ensured that the table was reliable and could be used for further analysis or decision-making.

### Statistical analysis

Data curation and statistical analysis were performed using Stata 17.0 (StataCorp LLC, Lake Drive, College Station, TX, USA). TDS and QAMAI scores for each domain and case were considered nonparametric data. Additionally, given the limited sample size, the median and interquartile range were used as descriptive statistics for continuous data.

Partial TDS scores were analysed individually before computing an overall TDS score. Similarly, the six QAMAI domains were analysed separately. The overall QAMAI score was generated by summing all domain scores and dividing the result by 6. All results were rounded to the nearest integer.

The TDS and QAMAI domain-specific scores from ChatGPT-4 and Gemini for each case were compared using the Wilcoxon signed-rank test. QAMAI domain-specific scores for each location were compared separately via the Friedman test for ChatGPT-4 and Gemini answers. Pairwise comparisons between subsites' performances were adjusted via Bonferroni correction. *P*-values less than 0.05 were considered statistically significant.

## Results

### Comparison of AI models to existing guidelines

Twelve prompts were collected between June 5 and 7, 2024. Of these, six originated from North America (four from the USA and two from Canada) and six from European countries (two each from Belgium, Switzerland, and Turkey) (Table 2). The prompt for Gemini originated in the USA (West Coast) generated three different tables (WC1, WC2, and WC3), so overall 14 tables were analysed. As both LLMs responded to the prompt (‘*a table of a bundle of care for perioperative neurocognitive disorders’*) by producing ‘recommendations’, and both guidelines presented a suite of ‘recommendations’, we used the term ‘recommendations’ to describe our results.

**Table 2:** Total Disagreement Score (TDS) and Quality Assessment of Medical Artificial Intelligence (QAMAI) score per large language model (LLM)—ChaptGPT-4 and Gemini—and per country. The TDS measures the level of disagreement between AI-generated care bundles and established guidelines, with scores ranging from 0 (complete agreement) to 3 (complete disagreement) across different management phases. The QAMAI score evaluates the AI outputs on six domains: accuracy, clarity, relevance, completeness, provision of sources, and usefulness, with scores ranging from 1 (strong disagreement) to 5 (strong agreement). Data are organised by country and date, reflecting the performance of each LLM in generating perioperative neurocognitive disorder care bundles.

Country and date	Canada June 7	Turkey June 7	Belgium June 6	USA (WC) 1 June 6	USA (WC) 2 June 6	USA (WC) 3 June 6	Switzerland June 5	USA (EC) June 6	Canada June 7	Turkey June 7	Belgium June 5	USA (WC) June 6	Switzerland June 5	USA (EC) June 6
LLM	Gemini								ChatGPT-4					
	TDS (0 agree to 3 disagree)													

**Preoperative management**	1	1	0	0	0	1	0	0	0	2	0	0	1	0
**Intraoperative management**	2	3	0	1	1	0	2	0	0	1	0	1	1	0
**Postoperative management**	0	1	1	1	1	1	0	1	1	1	1	1	1	1
**Other concerns**	0	0	0	0	0	0	0	0	0	0	0	0	0	0
**Total TDS**	3	5	1	3	2	2	2	1	1	4	1	2	3	1

	QAMAI (1 disagree to 5 agree)													

**Accuracy**	3	3	3	3	4	4	4	4	4	4	4	3	5	4
**Clarity**	4	3	3	4	4	5	4	4	4	4	4	3	5	4
**Relevance**	4	3	3	3	4	4	4	4	5	4	4	3	5	4
**Completeness**	4	3	3	3	4	5	4	4	5	4	4	3	5	4
**Provision of sources**	1	2	2	1	4	1	1	1	1	1	1	1	1	1
**Usefulness**	4	3	3	3	4	5	4	4	5	4	4	3	5	4

Overall complete agreement (TDS score of 0 and QAMAI score of 5 across all domains) was not achieved. The overall TDS were similar between both LLMs (2 [1–3] *vs* 2 [2–3], *P*=0.636; Supplementary material 2). Tables generated by ChatGPT-4 in Belgium, USA East Coast, and Canada and those generated by Gemini in Belgium and USA East Coast performed best with overall TDS scores of 1 (Table 2). Overall agreement for *preoperative* management was achieved in tables generated by Gemini in Belgium, USA (bi-coastal), and Switzerland, and by ChatGPT-4 in Belgium, USA (bi-coastal), and Canada. The highest disagreement was found in *intraoperative* management tables generated by Gemini in Turkey. *Postoperative* management showed overall agreement in tables generated by Gemini in Canada and Switzerland (Table 2).

The QAMAI scores were similar between both LLMs (4 [4–4] *vs* 4 [3–4], *P*=0.424; Supplementary material 2). The highest levels of agreement in accuracy, clarity, relevance, completeness, and usefulness were obtained for tables generated by ChatGPT-4 in Switzerland. Poorer levels of agreement were noted for tables generated by Gemini in Belgium, USA (West Coast), and by ChatGPT-4 in USA (West Coast). Regarding cited sources, total disagreement was observed for all tables except for one, the table generated by Gemini in USA (West Coast) (Table 2).

### Risk of bias assessment

Overall, 49 recommendations were coded, and 29 (59%) of those were derived from either the SBI or BHI recommendations.^10^, ^11^, ^12^, ^13^, ^14^ The LLMs generated the remaining 20 (41%) recommendations. The four most popular recommendations were: (1) ‘delirium monitoring: implement measures to monitor and detect delirium in the perioperative period’, with a score of 16/16; (2) ‘patients should be routinely screened postoperatively for these complications’, with a score of 15/16; (3) ‘risk should be disclosed to patients and families preoperatively’, with a score of 14.5/16; and (4) ‘postoperative pain: evaluate and effectively fine-tune preventive and postoperative pain treatment’, with a score of 14/16. All of these were included in the SBI and BHI recommendations.

The more complete recommendations were generated from ChatGPT-4 in Switzerland, adding up to 26 points, followed by ChatGPT-4 in Turkey (24 points) and ChatGPT-4 in Canada (23.5 points). The table with the smallest difference from the BHI recommendations was Switzerland (Gemini), with a total score of 11.5 and a difference of 2.0. The table that most resembled the SBI recommendations was Belgium (ChatGPT-4), with a total score of 13.0 and a difference of 9.5. The scores provided by Gemini and BHI did not differ significantly for the analysed recommendations (*P*>0.9). Based on the completeness and coverage of key measures, both ChatGPT-4 and Gemini appeared to provide comprehensive recommendations (*P*=0.844).

### Evaluation of their use in producing new guidelines

The top five most popular measures not included in the guidelines were: (1) early mobilisation (mentioned 13 times; 93%); (2) address modifiable risk factors (mentioned 12 times; 86%); (3) postoperative cognitive stimulation (mentioned 12 times; 86%); (4) maintain adequate blood pressure and oxygenation (mentioned 10 times; 71%); and (5) minimise brain ischaemia (mentioned seven times; 50%).

## Discussion

Our findings indicate that, although both ChatGPT-4 and Gemini LLMs can produce recommendations with a high degree of agreement with published guidelines, there are notable differences in their performance across various contexts. Specifically, the highest agreement and completeness were observed in recommendations generated by ChatGPT-4, suggesting that this AI model may offer potential enhancements over published guidelines by incorporating additional management strategies. The study found that Gemini's recommendations had the smallest difference from the BHI guideline,^11^^,^^14^ indicating a high level of alignment. Similarly, ChatGPT's recommendations closely matched the SBI guideline.^11^ These findings suggest that both LLMs can generate recommendations that align well with guidelines published by major societies, enhancing their credibility.

### Performance of AI models and clinical implications

Our study highlighted that, although 29 out of 49 recommendations were derived from the SBI or BHI guidelines, 20 recommendations were newly generated by LLMs. This contribution from LLMs emphasises their potential to enhance existing guidelines with additional, possibly innovative, insights. Notably, ChatGPT-4 produced the most comprehensive set of recommendations in Switzerland, accumulating 26 points, followed by 24 points in Turkey and 23.5 points in Canada. This suggests that ChatGPT-4 may have a slightly higher capability to generate thorough recommendations in certain regions.

Although overall agreement was not achieved, AI models generated recommendations not mentioned in the published guidelines.^11^, ^12^, ^13^, ^14^ Five measures—early mobilisation, addressing modifiable risk factors, postoperative cognitive stimulation, maintaining adequate blood pressure and oxygenation, and minimising brain ischaemia—were mentioned frequently across different AI-generated recommendations but are currently not included in the SBI or BHI guidelines. Their frequent occurrence highlights their perceived importance and suggests that these may be valuable additions to future guidelines. Future studies designed to produce specific evidence in PND prevention and treatment are essential to validate these AI-generated recommendations.

The concept of prehabilitation, which involves optimising a patient's state before an intervention, is well-known yet not routinely implemented.^34^ In this study, both the AI tools advocated for preoperative exercise for which benefits have been established^35^; however, guidelines have yet to incorporate this measure. Additionally, AI made other recommendations, such as early postoperative mobilisation, hydration, orientation strategies, and avoidance of restraints. These additions, not explicitly mentioned in any current PND guideline, but mentioned frequently in Enhanced Recovery After Surgery (ERAS) protocols, emphasise the need for a holistic patient-centred approach that incorporates both medical and environmental factors to minimise cognitive decline.

This study has several limitations. Only two LLMs were evaluated. All queries in this study were conducted in English, which may limit the generalisability of our findings to non-English-speaking contexts. Future research should explore the impact of multilingual prompts on AI-generated recommendations, as language-specific nuances may influence output quality and alignment with guidelines. The study's small scale and limited number of recommendations for PND may restrict the generalisability of the findings. A larger dataset and a broader spectrum of anaesthesia/non-anaesthesia recommendations are needed for a more comprehensive understanding of LLM response patterns to clinical scenarios. Geographic distribution was restricted to ratings from only five countries, potentially missing broader global variability. Each prompt was submitted to the AI models once, and only the initial response was considered. This approach may not capture the full variability and adaptability of AI responses. Modern generative AI systems using LLMs do not always provide consistent answers when prompted with the same medical question.^36^ This is because of their probabilistic next-word prediction nature. Evaluating multiple responses to the same prompt could provide a more thorough assessment of AI consistency and reliability, offering a better gauge of the range and quality of potential outputs. In contrast, rule-based systems, which can be human-designed or data-learned, are commonplace in medical practice and are guaranteed to provide the same answer for a given input. However, the power of modern generative AI systems lies exactly in their probabilistic nature. Another major limitation of AI-generated recommendations is the lack of references. Techniques such as Retrieval-Augmented Generation (RAG)^37^ enable proper source attribution, but this requires a large and domain-specific database (e.g. PubMed) to be used in addition to knowledge embedded in the LLM. Recently in 2024, Mija and colleagues^38^ have also cautioned against unvetted clinical application of general-purpose LLMs, commonly used by health practitioners, as these do not currently have default links to such domain-specific databases, although this is likely to change with further AI research.

The assessment tools, particularly the TDS scoring system, used to evaluate AI responses lack formal validation, which can introduce uncertainties in their effectiveness and reliability. Evaluation metrics such as TDS and QAMAI may not capture all relevant aspects of AI performance in clinical decision-making. However, these scores have been successfully used in a recent study on LLM performance in sinusitis cases.^31^ Moreover, subjective scoring by human evaluators introduces potential biases, affecting the consistency and reliability of the results. Gemini may confuse the reader by presenting three different recommendations when asked for one answer (USA, WC). The AI model presented three answers with significant differences.

The lack of cross-continental guidelines for PND care means there is no universally accepted standard of care, which may have influenced the evaluation of the AI-generated recommendations. Establishing cross-continental guidelines is crucial for interpreting the value of AI-generated recommendations, as they would provide a consistent benchmark against which their effectiveness and relevance can be measured. Developing and publishing clinical guidelines typically involves extensive research, multiple rounds of expert reviews, public consultations, and final approvals, often taking up to a year or more to complete. Additionally, SBI/BHI recommendations may have intentionally been limited to ensure conciseness or prioritise key evidence. In contrast, AI provides faster access to updated recommendations. However, the potential for hallucinations in LLMs must also be addressed. Although most LLM-generated recommendations aligned with published guidelines in our study, some novel suggestions, such as postoperative cognitive stimulation and minimising brain ischaemia, lacked strong clinical evidence. Implementing these without validation could pose risks. Additionally, the lack of source attribution makes it difficult to verify the reliability of these recommendations. Finally, the rapidly evolving nature of AI in medicine means that the study's findings may quickly become outdated.

### Future research

The AI-assisted generation of recommendations is an attractive option. Further research should explore the potential application of different AI models in various medical contexts to further elucidate their capabilities and limitations. Integrating clinician feedback to improve model outputs and exploring hybrid approaches that combine the strengths of both Gemini and ChatGPT-4 is of utmost importance. Moreover, although our study considered domain-agnostic AI models, another promising research direction is using domain-specific AI models such as Med-PaLM 2, which has been shown to outperform ChatGPT-4 on medical benchmarks.^37^ We note that access to these domain-specific AI models is often restricted. Open-source foundation models, with recent examples such as domain-agnostic Llama 3.1^39^ and medical-specific Meditron,^40^ enable almost anyone to use powerful modern LLMs. Integrating LLMs into medical practice may also help medical organisations or groups to develop recommendations by complementing and facilitating guideline creation. Newer versions of LLMs might also help improve their final output.

### Conclusions

Our findings indicate that ChatGPT-4 and Gemini can generate comprehensive and relevant recommendations that align closely with published guidelines. The frequent mention of measures not currently included in the guidelines suggests that LLMs can offer valuable additional insights and could be updated with recent literature. The high level of agreement and the comprehensive nature of the recommendations from both models highlight their potential utility in supporting clinical decision-making and enhancing guideline development. Although ChatGPT-4 and Gemini showed promising results, ChatGPT-4's outputs were noteworthy for their completeness and adherence to the included published guidelines. However, caution is warranted in the clinical application of AI-generated recommendations, and further research is needed to validate scoring systems and develop universally accepted guidelines. The integration of AI in perioperative care offers a promising avenue for improving patient outcomes, provided its limitations and ethical considerations are carefully managed. LLMs may generate recommendations that align with postoperative neurocognitive disorder guidelines. However, further validation and integration of clinician feedback are required before clinical application.

## Authors' contributions

Study design and conception: SS, OLBC, MS, BCM, SV, IM, MM, MG, JBE

Data acquisition, analysis, and interpretation: SS, OLBC, MS, BCM, SV, IM, MM, MG, JBE

Manuscript drafting: all authors

Manuscript revision critically for important intellectual content: all authors

Approval of the final version to be published: all authors

All authors agree to be accountable for all aspects of the work, thereby ensuring that questions related to the accuracy or integrity of any part of the work are appropriately investigated and resolved.

## Funding

National Institutes of Health, National Institute of General Medical Sciences award number K23GM132795 (to SV). Harold Amos Medical Faculty Development Program and Weill Award for Clinician Scientists in the Neurosciences (to OLBC).

## Declaration of Generative AI and AI-assisted technologies in the writing process

During the preparation of this work, the authors used ChatGPT 4.0 to improve readability. After using this tool/service, the authors reviewed and edited the content as needed and take full responsibility for the content of the publication.

## Declarations of interests

OLBC participated as an investigator for the clinical trial OLIVER from Medtronic. SS has received speaker's fees from Medtronic/Merck. JBE is a member of the Board of Directors of the European Society of Anesthesiology and Intensive Care (ESAIC) and has received speaker's fees from Medtronic.

## References

[1] Barreto Chang O.L., Possin K.L., Maze M. (2023). Age-related perioperative neurocognitive disorders: experimental models and druggable targets. Annu Rev Pharmacol Toxicol.

[2] Evered L., Silbert B., Knopman D.S. (2018). Recommendations for the nomenclature of cognitive change associated with anaesthesia and surgery-2018. Br J Anaesth.

[3] Meço B.C., Jakobsen K., De Robertis E. (2024). A first assessment of the safe brain initiative care bundle for addressing postoperative delirium in the postanesthesia care unit. J Clin Anesth.

[4] Saxena S., Maze M. (2018). Impact on the brain of the inflammatory response to surgery. Presse Med.

[5] Gleason L.J., Schmitt E.M., Kosar C.M. (2015). Effect of delirium and other major complications on outcomes after elective surgery in older adults. JAMA Surg.

[6] Evered L.A., Silbert B.S., Scott D.A., Maruff P., Ames D. (2016). Prevalence of dementia 7.5 years after coronary artery bypass graft surgery. Anesthesiology.

[7] Gou R.Y., Hshieh T.T., Marcantonio E.R. (2021). One-year medicare costs associated with delirium in older patients undergoing major elective surgery. JAMA Surg.

[8] Steinmetz J., Christensen K.B., Lund T., Lohse N., Rasmussen L.S. (2009). Long-term consequences of postoperative cognitive dysfunction. Anesthesiology.

[9] Barreto Chang O.L., Whitlock E.L., Arias A.D. (2023). A novel approach for the detection of cognitive impairment and delirium risk in older patients undergoing spine surgery. J Am Geriatr Soc.

[10] Hughes C.G., Boncyk C.S., Culley D.J. (2020). American society for enhanced recovery and perioperative quality initiative joint consensus statement on postoperative delirium prevention. Anesth Analg.

[11] Meço B.C., de Agua Reis A.B., Berger-Estilita J., Jakobsen K., Alkış N., Radtke F.M. (2023). Precision anaesthesia: advancing patient-centered precision care through repetitive assessment of PROMs with the Safe Brain Initiative approach. Turk J Anaesthesiol Reanim.

[12] Aldecoa C., Bettelli G., Bilotta F. (2024). Update of the European Society of Anaesthesiology and Intensive Care Medicine evidence-based and consensus-based guideline on postoperative delirium in adult patients. Eur J Anaesthesiol.

[13] Peden C.J., Miller T.R., Deiner S.G., Eckenhoff R.G., Fleisher L.A. (2021). Improving perioperative brain health: an expert consensus review of key actions for the perioperative care team. Br J Anaesth.

[14] Wooding D.J., Field T.S., Schwarz S.K.W. (2023). Current recommendations for perioperative brain health: a scoping review. J Neurosurg Anesthesiol.

[15] Bellman R. (1978).

[16] Ouyang L., Wu J., Jiang X. (2022). Training language models to follow instructions with human feedback. ArXiv.

[17] Henshall W. (2023). https://time.com/6300942/ai-progress-charts/.

[18] Cooper A., Rodman A. (2023). AI and medical education - a 21st-century pandora's box. N Engl J Med.

[19] Arango-Ibanez J.P., Posso-Nuñez J.A., Díaz-Solórzano J.P., Cruz-Suárez G. (2024). Evidence-based learning strategies in medicine using AI. JMIR Med Educ.

[20] Altamimi I., Alhumimidi A., Alshehri S. (2024). The scientific knowledge of three large language models in cardiology: multiple-choice questions examination-based performance. Ann Med Surg (Lond).

[21] Singam A. (2023). Revolutionizing patient care: a comprehensive review of artificial intelligence applications in anesthesia. Cureus.

[22] Ejikem M., Eya J., Ibu F. (2022). Perspectives of anesthesiologists towards the use of artificial intelligence in anesthesia practice in a developing country. J Anaesth Surg Res.

[23] Ayad S. (2023). Clinical applications of AI and machine learning in anesthesiology. ASA Monitor.

[24] Hashimoto D.A., Witkowski E., Gao L., Meireles O., Rosman G. (2020). Artificial intelligence in anesthesiology: current techniques, clinical applications, and limitations. Anesthesiology.

[25] Duran H.T., Kingeter M., Reale C., Weinger M.B., Salwei M.E. (2023). Decision-making in anesthesiology: will artificial intelligence make intraoperative care safer?. Curr Opin Anaesthesiol.

[26] World Medical Association (WMA) (2013). WMA declaration of Helsinki: Ethical principles for medical research involving human subjects. JAMA.

[27] von Elm E., Altman D.G., Egger M., Pocock S.J., Gøtzsche P.C., Vandenbroucke J.P. (2008). The Strengthening the Reporting of Observational Studies in Epidemiology (STROBE) statement: guidelines for reporting observational studies. J Clin Epidemiol.

[28] Tao Y., Viberg O., Baker R.S., Kizilcec R.F. (2024). Cultural bias and cultural alignment of large language models. PNAS Nexus.

[29] (2024). Welocalize. Adapting models to handle cultural variations in language and context. https://www.welocalize.com/insights/adapting-models-to-handle-cultural-variations-in-language-and-context/.

[30] Appen (2024). The pulse of language evolution. https://www.appen.com/blog/pulse-of-language-evolution.

[31] Saibene A.M., Allevi F., Calvo-Henriquez C. (2024). Reliability of large language models in managing odontogenic sinusitis clinical scenarios: a preliminary multidisciplinary evaluation. Eur Arch Otorhinolaryngol.

[32] Vaira L.A., Lechien J.R., Abbate V. (2024). Validation of the QAMAI tool to assess the quality of health information provided by AI. Eur Arch Otorhinolaryngol.

[33] Higgins J, Thomas J, Chandler J, *et al.* Cochrane Handbook for Systematic Reviews of Interventions version 6.4 2023. Available from: www.training.cochrane.org/handbook (accessed 7 July 2024).

[34] Gillis C., Ljungqvist O., Carli F. (2022). Prehabilitation, enhanced recovery after surgery, or both? A narrative review. Br J Anaesth.

[35] Feng X., Uchida Y., Koch L. (2017). Exercise prevents enhanced postoperative neuroinflammation and cognitive decline and rectifies the gut microbiome in a rat model of metabolic syndrome. Front Immunol.

[36] Singhal K., Tu T., Gottweis J. (2023). Towards expert-level medical question answering with large language models. ArXiv.

[37] Lewis P., Perez E., Piktus A. (2020). Retrieval-augmented generation for knowledge-intensive NLP tasks. Adv Neural Inf Process Syst.

[38] Mija D., Kehlet H., Rosero E.B., Joshi G.P. (2024). Evaluating the role of ChatGPT in perioperative pain management versus procedure-specific postoperative pain management (PROSPECT) recommendations. Br J Anaesth.

[39] Dubey A., Jauhri A., Pandey A. (2024). The Llama 3 herd of models. ArXiv.

[40] Chen Z., Cano AHa, Romanou A. (2023). MEDITRON-70B: scaling medical pretraining for large language models. ArXiv.

