# Supplementary material for: A comparison of large language model-generated and published perioperative neurocognitive disorder recommendations: a cross-sectional web-based analysis
Source: Br J Anaesth. 2025 Feb 7;136(4):1275–86. doi: 10.1016/j.bja.2025.01.001 (PMC13168983; doi:10.1016/j.bja.2025.01.001)
Supplement: Multimedia component 1 [file mmc1.docx]

**A comparison of large language model-generated and published perioperative neurocognitive recommendations: a cross-sectional web-based analysis: Supplement**

**Supplement 1 The SBI Bundle of Care^11^**

The SBI incorporates an extensive care bundle consisting of 18 core recommendations These recommendations primarily focus on non-invasive interventions aimed at detecting, preventing, and reducing adverse outcomes including postoperative delirium (POD), postoperative neurocognitive disorders (PND), postoperative nausea and vomiting (PONV), perioperative stress, perioperative anxiety, inadequate pain/nociception management, and patient discomfort. By implementing these recommendations, the SBI aims to address and mitigate potential complications and challenges associated with anaesthesia and surgery, ultimately improving patient outcomes and enhancing the overall surgical experience.


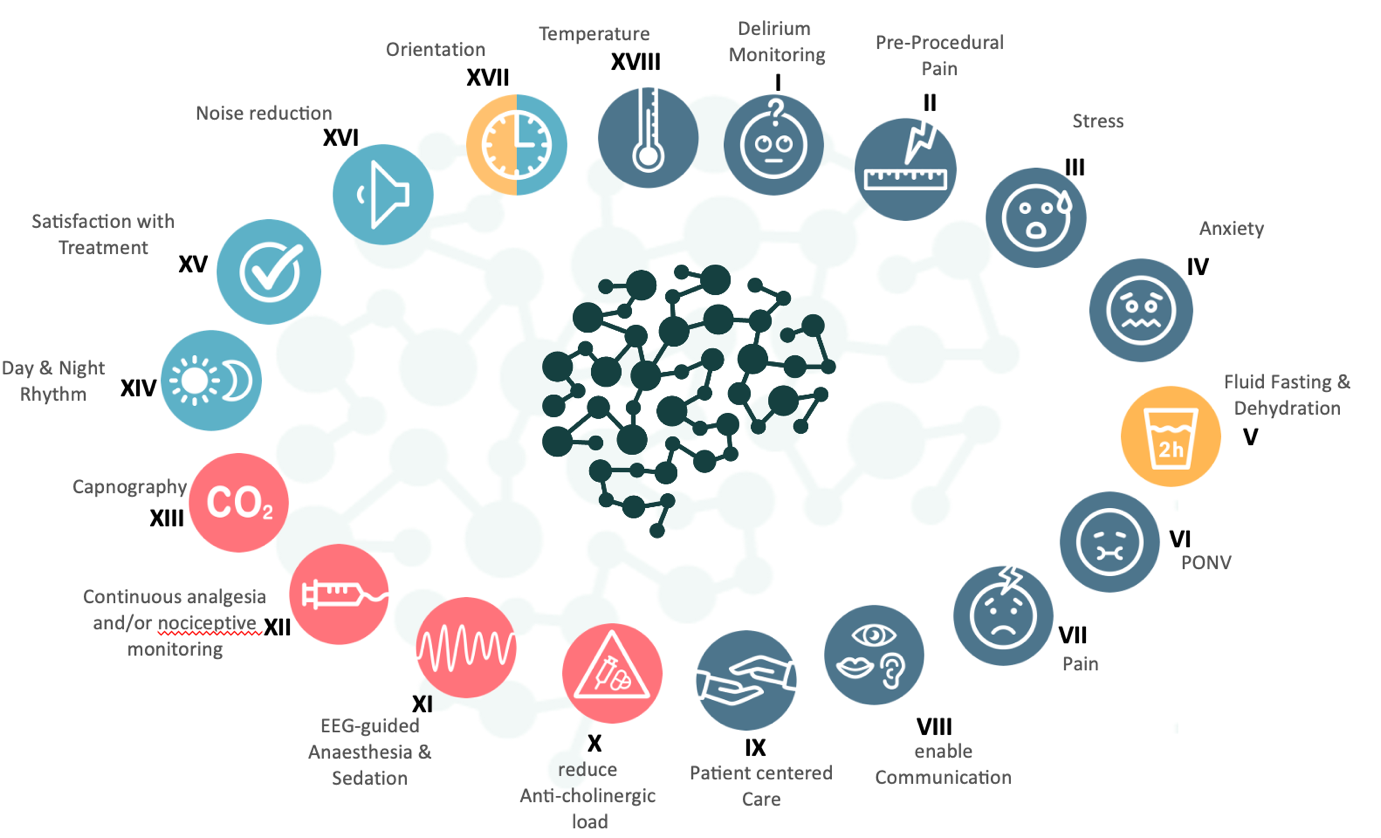


Figure 1: A multicomponent evidence-based approach: The 18 SBI Core recommendations

1. Delirium monitoring: Implement measures to monitor and detect delirium in the perioperative period.

2. Preoperative Pain: Address and treat pain before surgery to improve the overall patient experience.

3. Stress: Implement strategies to reduce perioperative stress and promote a more relaxed patient environment.

4. Anxiety: Recognize and address perioperative anxiety in patients through appropriate interventions and support.

5. Liquid fasting time: Reduce unnecessary fasting time before surgery.

6. Postoperative Nausea and Vomiting (PONV): Take steps to prevent and treat nausea and vomiting after surgery.

7. Postoperative Pain: Evaluate and effectively treat postoperative pain.

8. Communication: Improve communication between healthcare professionals and patients, ensuring a clear and effective exchange of information.

9. Patient-Centered Clinical Practice: Emphasize a patient-centred approach where the patient's preferences, needs, and values are considered and respected.

10. Anticholinergic influence: Consider the influence of anticholinergic medications on cognitive function and take steps to minimise their use where possible.

11. EEG Monitoring: Monitor the patient's brain activity using electroencephalography (EEG) to detect and prevent adverse neurological events.

12. Continuous analgesics (remifentanil): Use continuous analgesia techniques, such as remifentanil, to effectively manage pain during and after surgery.

13. Use of Capnography in sedated patients: Implement capnography under sedation to ensure adequate ventilation and detect possible complications.

14. Circadian Rhythm: Consider the patient's circadian rhythm and incorporate strategies to support the natural sleep-wake cycle during the perioperative period.

15. Patient Satisfaction: Measure and address patient satisfaction to improve the quality of care provided continually.

16. Noise: Minimize noise levels in the perioperative environment to promote a calmer and more comfortable patient atmosphere.

17. Orientation: Ensure patients are properly oriented in space and time during the perioperative period.

18. Temperature: Maintain proper perioperative temperature management to prevent hypothermia or hyperthermia and promote patient comfort.

The interventions recommended by the Safe Brain Initiative (SBI) are designed to alleviate the iatrogenic burden on patients' postoperative outcomes. These recommendations are derived from international guidelines and aim to enhance the patient's perioperative experience, minimise complications, and promote positive outcomes. By implementing these non-invasive measures, healthcare providers can contribute to safer, more patient-centered perioperative care. The SBI recognises the importance of aligning interventions with evidence-based guidelines to ensure optimal outcomes and improve the overall quality of care for patients undergoing anaesthesia and surgery.

**Supplement 2: The BHI bundle of care^14^**

**^
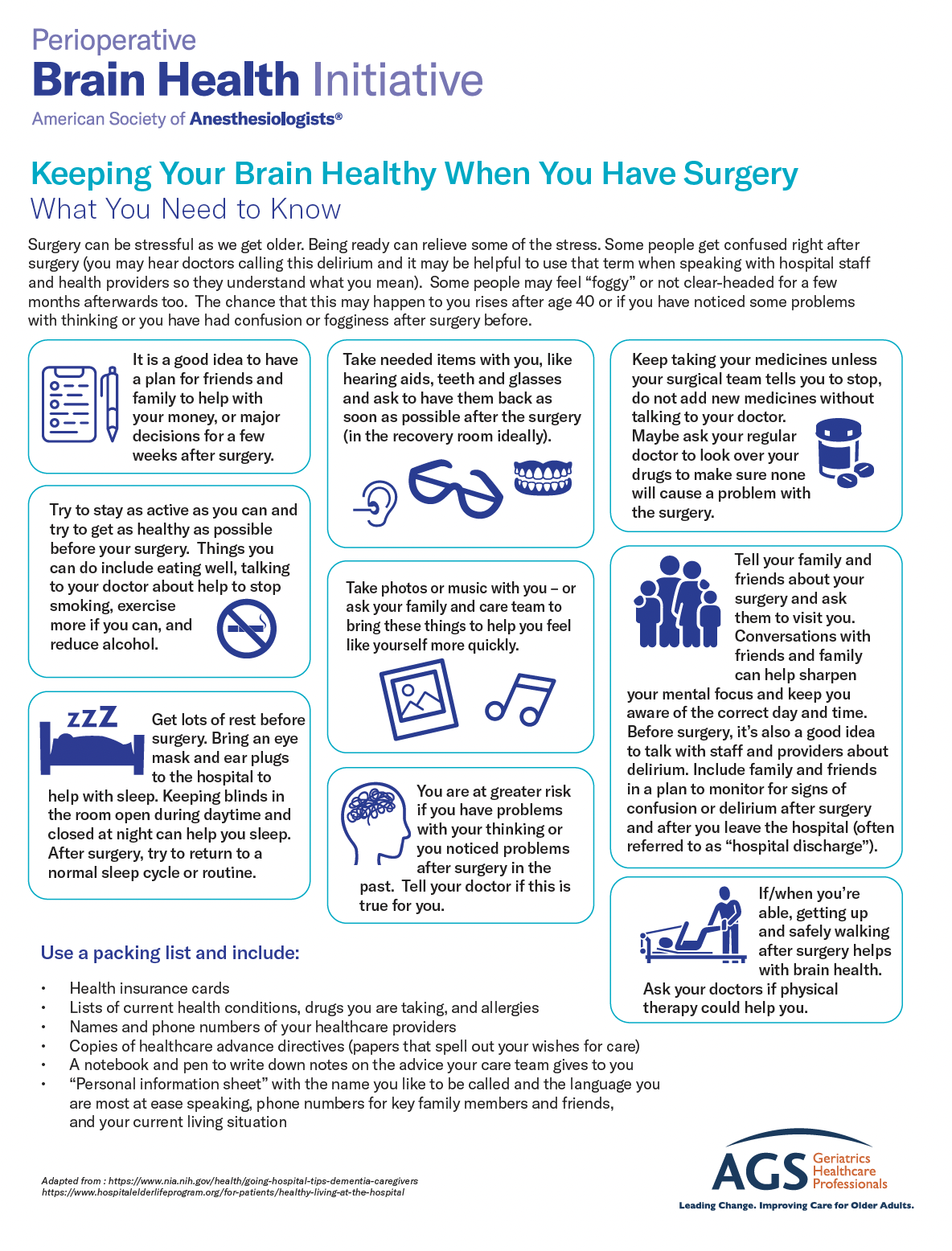
^**

*reproduced with permission ^14^*

Supplement eTable 1:

**Overall and partial ratings for the total disagreement score (TDS) and Overall and domain-specific quality assessment of medical artificial intelligence (QAMAI) ratings**

|  | ChatGPT 4 (N=6) | Gemini (N=8) | *P* value |
| --- | --- | --- | --- |
| Pre-operative management (median; Q1:Q3) | 0 (0:1) | 0 (0:1) | 0.939 |
| Intra-operative management (median; Q1:Q3) | 1 (0:1) | 1 (0:2) | 0.301 |
| Post-operative management (median; Q1:Q3) | 1 (1:1) | 1 (1:1) | 0.202 |
| Other concerns (median; Q1:Q3) | 0 (0:0) | 0 (0:0) | > 0.999 |
| Total score (median; Q1:Q3) | 2 (1:3) | 2 (2:3) | 0.636 |
| Accuracy (median; Q1:Q3) | 4 (4:4) | 4 (3:4) | 0.142 |
| Clarity (median; Q1:Q3) | 4 (4:4) | 4 (4:4) | 0.705 |
| Relevance (median; Q1:Q3) | 4 (4:5) | 4 (3:4) | 0.147 |
| Completeness (median; Q1:Q3) | 4 (4:5) | 4 (3:4) | 0.293 |
| Provision of sources (median; Q1:Q3) | 1 (1:1) | 1 (1:2) | 0.105 |
| Usefulness (median; Q1:Q3) | 4 (4:5) | 4 (3:4) | 0.293 |
| Overall (median; Q1:Q3) | 4 (4:4) | 4 (3:4) | 0.424 |
