# Supplementary material for: A comparison of large language model-generated and published perioperative neurocognitive disorder recommendations: a cross-sectional web-based analysis
Source: Br J Anaesth. 2025 Feb 7;136(4):1275–86. doi: 10.1016/j.bja.2025.01.001 (PMC13168983; doi:10.1016/j.bja.2025.01.001)
Supplement: Multimedia component 2 [file mmc2.docx]

**Supplementary Digital File 2**

**eTable1: Overall and partial ratings for the total disagreement score (TDS) and Overall and domain-specific quality assessment of medical artificial intelligence (QAMAI) ratings**

|  | ChatGPT 4 (N=6) | Gemini (N=8) | *P* value |
| --- | --- | --- | --- |
| Pre-operative management (median; Q1:Q3) | 0 (0:1) | 0 (0:1) | 0.939 |
| Intra-operative management (median; Q1:Q3) | 1 (0:1) | 1 (0:2) | 0.301 |
| Post-operative management (median; Q1:Q3) | 1 (1:1) | 1 (1:1) | 0.202 |
| Other concerns (median; Q1:Q3) | 0 (0:0) | 0 (0:0) | > 0.999 |
| Total score (median; Q1:Q3) | 2 (1:3) | 2 (2:3) | 0.636 |
| Accuracy (median; Q1:Q3) | 4 (4:4) | 4 (3:4) | 0.142 |
| Clarity (median; Q1:Q3) | 4 (4:4) | 4 (4:4) | 0.705 |
| Relevance (median; Q1:Q3) | 4 (4:5) | 4 (3:4) | 0.147 |
| Completeness (median; Q1:Q3) | 4 (4:5) | 4 (3:4) | 0.293 |
| Provision of sources (median; Q1:Q3) | 1 (1:1) | 1 (1:2) | 0.105 |
| Usefulness (median; Q1:Q3) | 4 (4:5) | 4 (3:4) | 0.293 |
| Overall (median; Q1:Q3) | 4 (4:4) | 4 (3:4) | 0.424 |
